# Repurposing Study of 4-Acyl-1-phenylaminocarbonyl-2-substituted-piperazine Derivatives as Potential Anticancer Agents—In Vitro Evaluation against Breast Cancer Cells

**DOI:** 10.3390/ijms242317041

**Published:** 2023-12-01

**Authors:** Emilio Guillén-Mancina, María del Rosario García-Lozano, Estefanía Burgos-Morón, Sarah Mazzotta, Pablo Martínez-Aguado, José Manuel Calderón-Montaño, José Manuel Vega-Pérez, Miguel López-Lázaro, Fernando Iglesias-Guerra, Margarita Vega-Holm

**Affiliations:** 1 Department of Pharmacology, Faculty of Pharmacy, University of Seville, 41012 Seville, Spain; eguillen@us.es (E.G.-M.); eburgos1@us.es (E.B.-M.); jcalderon@us.es (J.M.C.-M.); mlopezlazaro@us.es (M.L.-L.); 2 Department of Organic and Medicinal Chemistry, Faculty of Pharmacy, University of Seville, 41012 Seville, Spain; mariadelrosariogarcialozano@gmail.com (M.d.R.G.-L.); sarah.mazzotta@unimi.it (S.M.); pamasoy@hotmail.com (P.M.-A.); vega@us.es (J.M.V.-P.); 3 Institute of Biomedicine of Seville (IBiS), Virgen del Rocío University Hospital, CSIC, University of Seville, 41013 Seville, Spain; 4 Department of Chemistry, University of Milan, 20133 Milan, Italy; 5 Infectious Diseases and Microbiology Clinical Unit, University Hospital Virgen Macarena, 41009 Seville, Spain; 6 Departament of Medicine, School of Medicine, University of Seville, 41012 Seville, Spain

**Keywords:** breast cancer, cellular viability, piperazine, aryl urea, selective cytotoxic activity

## Abstract

Breast cancer is the most common type of cancer in women. Although current treatments can increase patient survival, they are rarely curative when the disease is advanced (metastasis). Therefore, there is an urgent need to develop new cytotoxic drugs with a high selectivity toward cancer cells. Since repurposing approved drugs for cancer therapy has been a successful strategy in recent years, in this study, we screened a library of antiviral piperazine-derived compounds as anticancer agents. The compounds included a piperazine ring and aryl urea functions, which are privileged structures present in several anti-breast cancer drugs. The selective cytotoxic activity of a set of thirty-four 4-acyl-2-substituted piperazine urea derivatives against MCF7 breast cancer cells and MCF 10A normal breast cells was determined. Compounds **31**, **32**, **35**, and **37** showed high selective anticancer activity against breast cancer cells and were also tested against another common type of cancer, non-small cell lung cancer (A549 lung cancer cells versus MRC-5 lung normal cells). Compounds **35** and **37** also showed selectivity against lung cancer cells. These results suggest that compounds **35** and **37** may be promising hit compounds for the development of new anticancer agents.

## 1. Introduction

With over 2.3 million new cases estimated, breast cancer was the most prevalent cancer diagnosed worldwide at the end of 2020 [1]. By 2040, the burden of breast cancer is estimated to increase to more than 3 million new cases and 1 million deaths every year due to the growth and aging of the population [2]. Most deaths are caused by metastasis, that is, when cancer cells have spread to other tissues and organs [3]. Although pharmacotherapy, the main form of treatment for patients with metastases, can prolong patient survival, it is not enough to cure the cancer in most cases. Most current anticancer drugs have narrow selectivity toward cancer cells, and they also damage healthy cells, especially those with a high proliferative rate, such as cells of the immune system. Therefore, anticancer drugs are usually used in doses that are tolerable but not enough to kill all cancer cells, and the disease progresses to death. New drugs that improve the efficacy of current antitumor therapies for breast cancer are urgently needed. Medicinal chemists play an important role in the discovery of anticancer agents through the design, optimization, and development of new chemical compounds [4,5,6,7].

**Figure 1:**
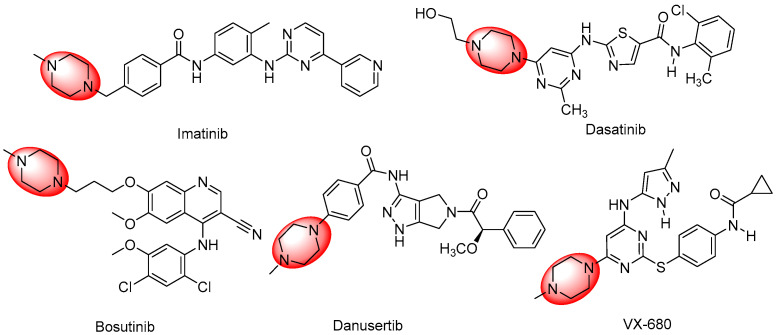
Structures of anticancer drugs containing piperazine ring highlighted in red.

The piperazine scaffold represents a very important class of bioactive N-heterocyclic compounds due to their pharmacological versatility being widely used in research for the development of new molecules for diverse therapeutic targets. It has been reported that a wide range of biological applications of molecules containing piperazine are an essential subunit of the structural frame. These include antibacterials and antifungals [8,9,10,11,12], antiviral [13,14,15,16], antipsychotic [17], antihypertensive [18], and antioxidant [19], among others. Many currently notable anticancer drugs, such as imatinib (STI571) [20], dasatinib (BMS-354825) [21], bosutinib (SKI-606) [22], danusertib (PHA-739358) [23], and VX-680 [24], contain a piperazine ring as part of their molecular structure (Figure 1).

**Figure 2:**
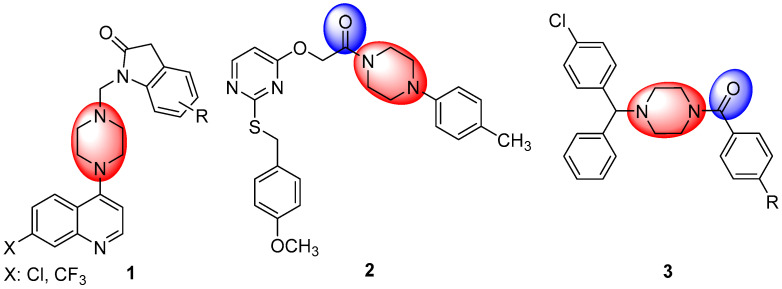
General structures of piperazine-derived compounds **1**–**3** showing acyl group and piperazine moiety in blue and red colors, respectively.

In the literature, novel piperazine derivatives or skeletons containing piperazine have been described as antiproliferative agents against breast cancer (Figure 2) [25,26,27]. Hybrid compounds containing pharmacophores with 4-piperazinyl-quinoline-isatin derivatives (**1**) exhibited cytotoxicity against two human breast cancer cell lines (MDA-MB468 and MCF7), with IC_50_ values in the range of 10.34–66.78 μM [25]. Three of the compounds tested showed two- to four-fold lower cytotoxic effects in noncancerous breast cell lines (184B5 and MCF 10A). Thiouracil amide derivatives bearing a piperazine ring (**2**) were synthesized and screened for cytotoxic activity in MCF7 cells, with IC_50_ values in the range of 18.23 to 100 µM [26]. The most active compounds, those with tolyl or halophenyl group alkylating the piperazine ring, were two to three times less cytotoxic against normal MCF 10A cells than cancer cells. Also, small molecules such as 1-(4-substitutedbenzoyl)-4-(4-chlorobenzhydryl) piperazine derivatives (**3**) with different groups at the aryl amide function showed cytotoxic activity against breast cancer cells (MCF7, BT20, T47D, and CAMA-1) and other types of cancer cell lines [27]. These compounds were also evaluated in normal breast epithelial cells (MCF-12A). The IC_50_ values were in the range of 0.31–120.52 μM against breast cancer cell lines and 6.6–299.66 μM against normal breast cells.

**Figure 3:**
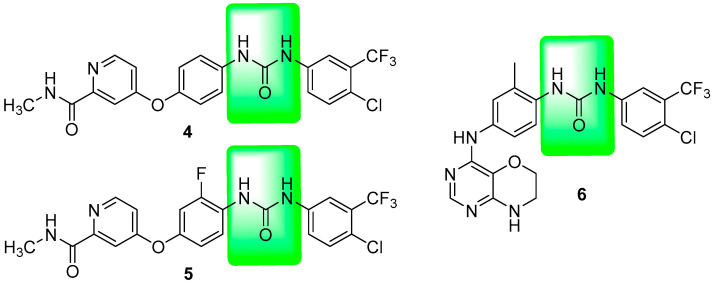
Structures of biaryl-urea kinase inhibitors with the urea function highlighted in green.

The urea function also constitutes a relevant moiety in anticancer agents. Biaryl-urea-based kinase inhibitors have been approved in anticancer therapy, for example, sorafenib (**4**) and its analog, regorafenib (**5**). The development of novel biaryl-urea-based small molecules as potential kinase inhibitors has been increasingly highlighted with the clinical success of these compounds [28], such as compound **6**, a multikinase inhibitor that showed significant anticancer activity against diverse cancer cells [29] (Figure 3).

Drug repurposing or repositioning means establishing new medical uses for already-known drugs. Although this is not a new strategy, it has become more popular in recent years, reaching around 25% of the annual pharmaceutical industry revenue. In the past decades, several drugs that were originally approved for indications other than cancer treatment have shown antitumoral effects [30,31].

The objective pursued in this study was to screen a library of antiviral piperazine-derived compounds for their possible repositioning as anticancer agents since piperazine ring and aryl urea functions are privileged structures present in anticancer drugs.

## 2. Results and Discussion

**Figure 4:**
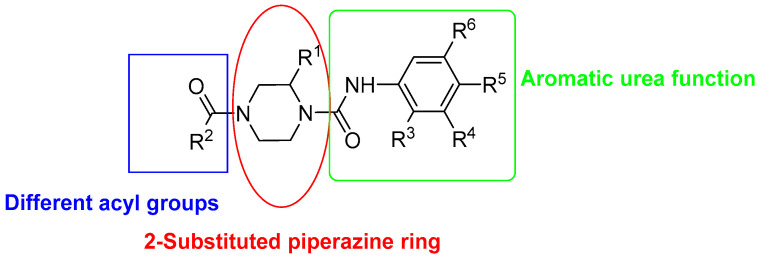
General structure of 4-acyl-2-substituted piperazine urea derivatives.

In this work, we report the biological evaluation of a set of 34 compounds 4-N-acyl-1-phenylamino(thio)carbonyl-2-substituted piperazine derivatives (previously described as effective antiadenovirus agents) [15] against breast cancer cells. Figure 4 shows the general scaffold of our compounds that consists of a piperazine ring as a central core with a phenylamino(thio)carbonyl group at nitrogen 1 and different acyl groups at nitrogen 4.

Our general skeleton has three points of structural variation: the substituent on the piperazine ring (R^1^: methyl or phenyl), the acyl group at N-4 (R^2^: pivaloyl, benzoyl, *tert*-butoxycarbonyl, or benzofuran-2-carbonyl) and the different substituents at the phenyl ring in the urea function (electron-withdrawing or electron-releasing groups).

The collection of compounds **7**–**39** is shown in Table 1.

### 2.1. Evaluation of Selective Cytotoxic Activity of 4-Acyl-2-substituted Piperazine Urea Derivatives against MCF7 Breast Cancer Cells and MCF 10A Normal Breast Cells

**Figure 5:**
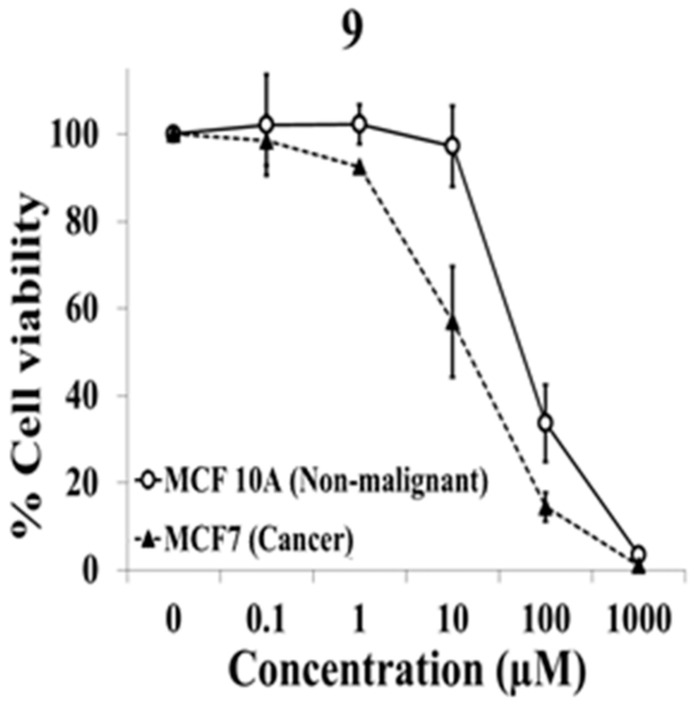
Evaluation of cytotoxic activity of compound **9** on human nonmalignant breast cells (MCF 10A) and human breast cancer cells (MCF7). Cells were exposed to several concentrations of compound for 72 h, and cell viability was determined with the MTT assay. Data represent mean ± SE) from at least two independent experiments.

**Figure 6:**
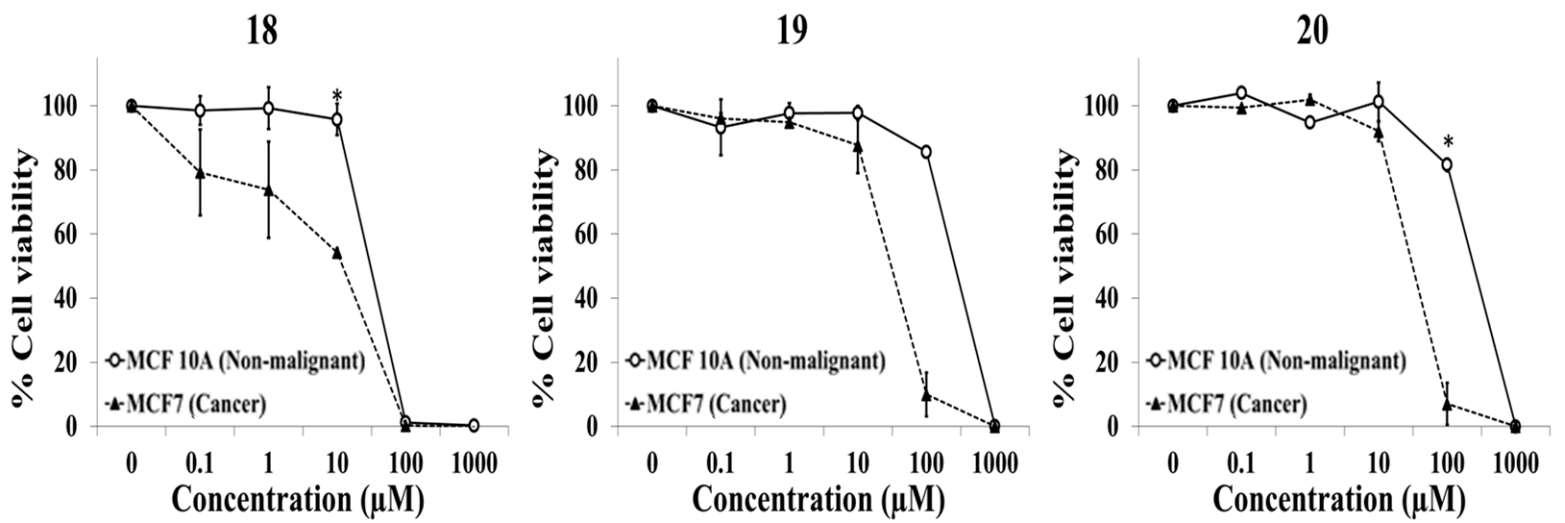
Evaluation of cytotoxic activity of compounds **18**–**20** on human nonmalignant breast cells (MCF 10A) and human breast cancer cells (MCF7). Cells were exposed to several concentrations of compounds for 72 h, and cell viability was determined with the MTT assay. Data represent mean ± SEM from at least two independent experiments. Student’s *t*-test was performed to compare the cytotoxicity of a particular concentration of the compound between MCF 10A and MCF7. * indicates *p* ˂ 0.05.

**Figure 7:**
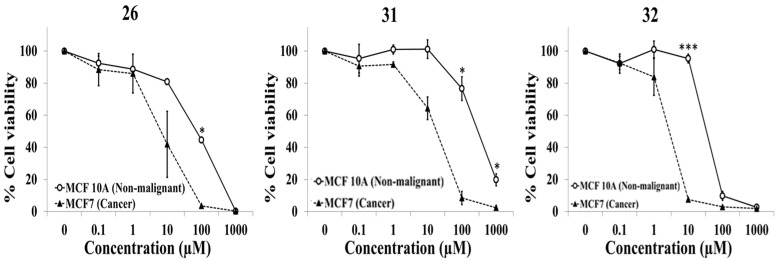
Evaluation of cytotoxic activity of compounds **26**, **31**, and **32** on human nonmalignant breast cells (MCF 10A) and human breast cancer cells (MCF7). Cells were exposed to several concentrations of compounds for 72 h, and cell viability was determined with the MTT assay. Data represent mean ± SEM from at least two independent experiments. Student’s *t*-test was performed to compare the cytotoxicity of a particular concentration of the compound between MCF 10A and MCF7. * indicates *p* ˂ 0.05, *** indicates *p* ˂ 0.001.

**Figure 8:**
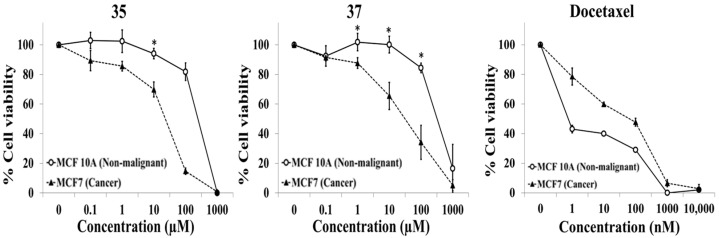
Evaluation of cytotoxic activity of compounds **35**, **37**, and docetaxel on human nonmalignant breast cells (MCF 10A) and human breast cancer cells (MCF7). Cells were exposed to several concentrations of compounds for 72 h, and cell viability was determined with the MTT assay. Data represent mean ± SEM from at least two independent experiments. Student’s *t*-test was performed to compare the cytotoxicity of a particular concentration of the compound between MCF 10A and MCF7. * indicates *p* ˂ 0.05.

**Figure 9:**
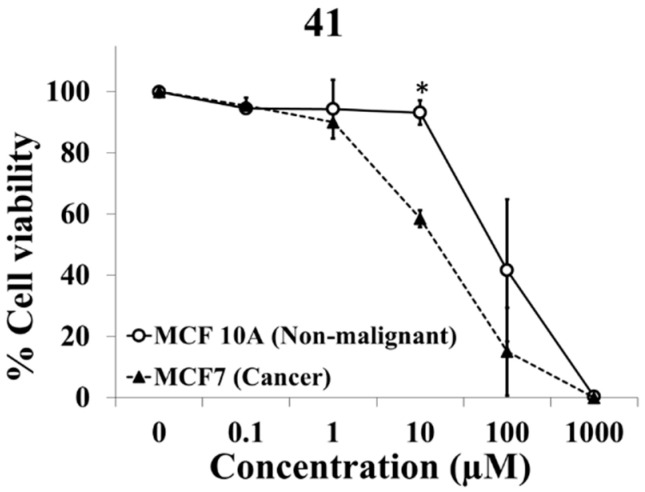
Evaluation of cytotoxic activity of compound **41** on human nonmalignant breast cells (MCF 10A) and human breast cancer cells (MCF7). Cells were exposed to several concentrations of compounds for 72 h, and cell viability was determined with the MTT assay. Data represent mean ± SEM from at least two independent experiments. Student’s t-test was performed to compare the cytotoxicity of a particular concentration of the compound between MCF 10A and MCF7. * indicates *p* ˂ 0.05.

To evaluate the cytotoxicity of our compounds against cancer cells, they were evaluated in vitro against MCF7 human breast cancer cells. To examine the selectivity of our compounds, they were also studied against MCF 10A human normal cells. Both cell lines were exposed to different concentrations of our compounds for 72 h before measuring cell viability with the 3-(4,5-dimethylthiazol-2-yl)-2,5-diphenyltetrazolium bromide (MTT) assay (for conditions of the MTT assay, see the Experimental Part, Section 3). Docetaxel, an anticancer drug used in clinics [32], was tested under the same experimental conditions. Data were obtained from at least two independent experiments and were expressed as means ± standard error of means (SEM). The half-maximal inhibitory concentration (IC_50_) value of each compound was calculated, and the results are collected in Table 2. To evaluate the selective activity of our compounds, the selectivity index (SI) was also calculated. The curves of cell viability of MCF7 cancer cells and MCF 10A normal cells after the treatment with our compounds are shown (Figure 5, Figure 6, Figure 7, Figure 8 and Figure 9 and Figures S1–S4).

### 2.2. Structure Activity Relationship

In order to obtain structural information about which organic function contributes to the biological activity, firstly, the methyl piperazine ring was selected as the central core, and chemical diversity was focused on N-4 by the presence of three different acyl groups (pivaloyl, benzoyl, and *tert*-butoxycarbonyl). NO_2_ and OMe (at *para* position of the phenyl ring in the urea) were selected as prototypes of groups with both electronic properties mentioned above. See Table 2 for the biological data mentioned in the discussion.

Compounds **7**, **10**, and **12** (pivaloyl, benzoyl, and *tert*-butoxycarbonyl derivatives, respectively) with a 4-NO_2_ group at the phenyl ring in the urea function were more active than their analogs with a 4-OMe group (**8**, **11**, and **13** respectively), which showed higher IC_50_ values against MCF7 cancer cells (Figure 5 and Figure S1). Among the NO_2_ derivatives, **10** (with an aromatic amide at N-4) gave the lowest IC_50_ (26.7 µM). An electron-withdrawing group is important for the activity in this type of compound. However, compounds **7**, **10**, and **12** proved to be less selective against cancer cells than their analogs.

Two modifications were present in the analogs that allowed us to study their effect on anticancer activity.

Firstly, the isosteric modification urea by thiourea. For those 4-NO_2_ urea derivatives, **7** and **12**, their thiourea analogs were studied (Figure 5 and Figure S1). Compound **9** (pivaloyl thiourea derivative) was over four times more cytotoxic against cancer cells than **7** (the IC_50_ values were, respectively, 15.8 µM and 60.8 µM) and showed higher anticancer selectivity. However, this modification did not improve the activity in compound **14** (*tert*-butoxycarbonyl thiourea derivative, IC_50_ 34.2 µM vs. 34.6 µM for **12**).

Secondly, the replacement of the NO_2_ group at the phenyl ring in compound **12** by different electron-withdrawing groups was considered. Compounds **15**, 4-Cl, **16**, 4-CF_3_, and **17**, 3-CF_3_, 4-Cl, were evaluated (Figure S1). Their IC_50_ values were 43.4 µM, 31.3 µM, and 22.7 µM, respectively, against the cancer cell line. Compound **17** (with two groups in the aromatic ring, 3-CF_3_, 4-Cl) was the one that presented a lower IC_50_ value than **12** (22.7 µM vs. 34.6 µM). Additionally, they also showed slight selectivity against cancer cells (SI range 1.9–3.8).

Due to the benzofuran ring being an important privileged structure for anticancer activity compounds [33], the 2-methylpiperazines (same central core) having a 2-benzofuran-2-carbonyl at N-4 were also evaluated (Figure 6 and Figure S2). A set of different electron-withdrawing groups in the phenyl ring in the urea function were considered mainly in *para* position but also in *ortho* position. In general terms, the benzofuran-2-carbonyl derivatives with an electron-withdrawing group at *para* position were more active against the cancer cell line than those with other acyl groups at N-4, giving lower IC_50_ values (compound **18,** 4-NO_2_, 12.1 µM; compound **19**, 4-Cl, 30.5 µM; compound **20**, 4-CF_3_, 31.7 µM; and compound **21**, 4-CN, 19.4 µM). Related to the presence of the group in *ortho* position, it did not optimize the activity. The 2-NO_2_ **22**, 2-OCH_3_ **23**, and 2-Br **24** derivatives showed similar IC_50_ (25.3–31.4 µM). Compound **25**, with an unsubstituted phenyl ring in the urea, gave the highest IC_50_ value (48.5 µM). From these screenings, it can be concluded that the benzofurane ring and an electron-withdrawing group at *para* position in the phenyl of the urea are structural features linked to both nitrogens of the 2-methyl piperazine core that contribute to increased anticancer activity.

It is important to note that the selective anticancer effects of the compounds remain an issue to be addressed. For the most active derivatives, **9**, **18**, and **21**, with IC_50_ < 20 µM, the highest SI was 4.2 (compound **9**). Compounds **19** and **20** showed higher SI, being approximately eight times more toxic to cancer cells than normal cells; however, these compounds display selective anticancer activity at concentrations within a narrow range (Figure 6).

At this point, we were focused on increasing the anticancer activity and on reducing the toxicity. Since the replacement of the 2-methyl piperazine core by 2-phenyl piperazine led to more active and noncytotoxic antiviral compounds [15], this backbone was also present in the tested compounds. *tert*-Butoxycarbonyl and benzofuran-2-carbonyl were the two acyl moieties at N-4.

Compounds **26**–**29**, N-4-*tert*-butoxycarbonyl-2-phenyl piperazine derivatives, were screened (Figure 7 and Figure S3). These compounds possessed electron-withdrawing groups at the urea function, a structural feature found to be crucial for anticancer activity. Three compounds showed IC_50_ values < 20 µM against the cancer cell line (**26**, 4-NO_2_, IC_50_ 9.6 µM; **27**, 4-Cl, IC_50_ 16.8 µM; **28**, 4-CN, IC_50_ 19.5 µM), while the 4-F derivative **29** showed higher IC_50_ (24.8 µM). To evaluate whether the requirements for improving the activity were like the 2-methyl piperazine analogs, the 2-NO_2_ derivative (**30**) as well as two electron-donating groups in *para* position, OCH_3_ (**33**) and CH_3_ (**34**), were also evaluated (Figure 7 and Figure S3), showing higher IC_50_ values (24.3 µM, 130.4 µM, and 24.4 µM, respectively), which supports the need for an electron-withdrawing group in *para* position. Two disubstituted derivatives were also tested, **31** (2-Cl, 5-CF_3_) and **32** (3-CF_3_, 4-Cl), showing IC_50_ data of 18.6 µM and 2.7 µM, respectively. The substitution pattern in **32** was like **17**, its 2-methyl piperazine analog, but more active (2.7 µM vs. 22.7 µM), and like those biarylurea-based compounds mentioned above (Figure 7 and Figure S1).

Related to the selective profile, the SI values for these 2-phenyl-piperazine derivatives also increased, highlighting two compounds (**31** and **32**) with 16.6 and 12.6 values, respectively.

The last structural consideration was the presence of the benzofuran-2-carbonyl group at N-4, keeping electron-withdrawing groups at the urea. Compounds with 4-NO_2_ (**35**), 4-Cl (**36**), 4-CN (**37**), 2-Cl,5-CF_3_ (**38**), and 2-NO_2_ (**39**) were less cytotoxic against MCF7 cancer cells (Figure 8 and Figure S4) than their Boc analogs. The benzofuran-2-carbonyl analogs showed higher IC_50_ (23.0 µM, 41.3 µM, 57.1 µM, 91.9 µM, and 225.9 µM, respectively). In general terms, the Boc 2-phenyl derivatives presented a better cytotoxic activity profile (lower IC_50_) than the corresponding benzofuran-2-carbonyl analogs (**26** vs. **35**, **27** vs. **36**, **28** vs. **37**, and **31** vs. **38**) (Figure 7, Figure 8, Figures S3 and S4).

The comparison of both types of piperazine core showed that the 2-phenylpiperazine skeleton generated compounds with higher SI, showing less cytotoxicity against normal cells than the 2-methyl derivatives.

From this collection (Table 1), based on the experimental data described, five Boc 2-phenyl derivatives with IC_50_ < 20 µM were chosen as potential agents against breast cancer: **26**, **27**, **28**, **31**, and **32**. They were submitted for in silico evaluation of their physicochemical and pharmacokinetic properties. **31** and **32** resulted the most promising compounds, with better selective anticancer activity compared with docetaxel (Table 2). Docetaxel is a common anticancer agent used in the treatment of solid tumors, including breast cancer. However, this drug induces numerous adverse effects because it also affects normal tissues. Under our experimental conditions, MCF 10A normal cells were more sensitive to docetaxel than MCF7 cancer cells (SI < 1). Similar results have been reported by other authors [34,35]. In contrast, compounds **31** and **32** were more cytotoxic on MCF7 cancer cells than MCF 10A normal cells.

Having in mind the fact that the presence of the benzofuran-2-carbonyl group at N-4 in the 2-methyl piperazine core gave better activity data than the Boc group (lower IC_50_ values were observed), we decided to preserve both characteristics and replace the aryl group at the urea function. We prepared a conjugate of these two-membered scaffolds with adamantane moiety, also employing the urea function as a linker connecting the molecular fragments. We chose this polycyclic cage because it has been described as a privileged structure in promising anticancer agents [36,37,38], also present in hybrids with other active compounds such as colchicine (an ester group was the linker connector), revealing high cytotoxicity to cancer cells [39].

**Scheme 1:**
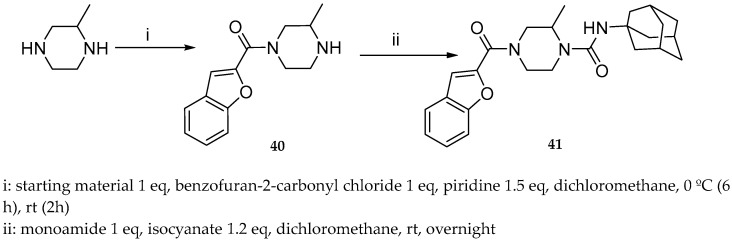
Synthesis of compound **41**.

The synthetic methodology was a brief and high-yielded route, previously described, that consists of two reactions (Scheme 1) [15,16]. The first step involved the introduction of the benzofuran-2-carbonyl group through a chemoselective N-acylation reaction of 2-methyl piperazine with the appropriate acyl chloride reactive providing the introduction of the amide function at the less hindered nitrogen (**40**). From this monoacyl derivative, the urea group was introduced at the other nitrogen by reaction with adamantyl isocyanate. Compound **41** was obtained in high yield. Spectral (nuclear magnetic resonance (NMR) and mass spectrometry) and analytical data of **41** showed full agreement with the proposed structure (see Experimental Part, Section 3, Figures S5 and S6, Supplementary Information).

Under the same experimental conditions, compound **41** also showed cytotoxicity against the MCF7 breast cancer cell line and selective anticancer activity (Figure 9). MCF7 cancer cells were ~5 times more sensitive to compound **41** than MCF 10A normal cells, with IC_50_ values (mean ± SEM; µM) of 17.7 ± 5.0 and 102.7 ± 66.9, respectively. SI (mean ± SEM) was 5.1 ± 2.3. Compound **41** was also selected for in silico evaluation of drug-likeness properties.

In summary, among the thirty-four compounds examined, seven compounds showed high cytotoxic activity against breast cancer cells, with IC_50_ ranging from 12.1 to 19.5 µM, and two showed IC_50_ values of <10 µM (2.7 and 9.6 µM). Four compounds (**31**, **32**, **35**, **37**) were >10 times more cytotoxic to cancer cells than to normal cells, being the most selective anticancer compounds.

### 2.3. Evaluation of the Selective Cytotoxic Activity of 4-N-acyl-1-phenylamino(thio)carbonyl-2-substituted Piperazine Derivatives against Nonmalignant Cells and Lung Cancer Cells

**Figure 10:**
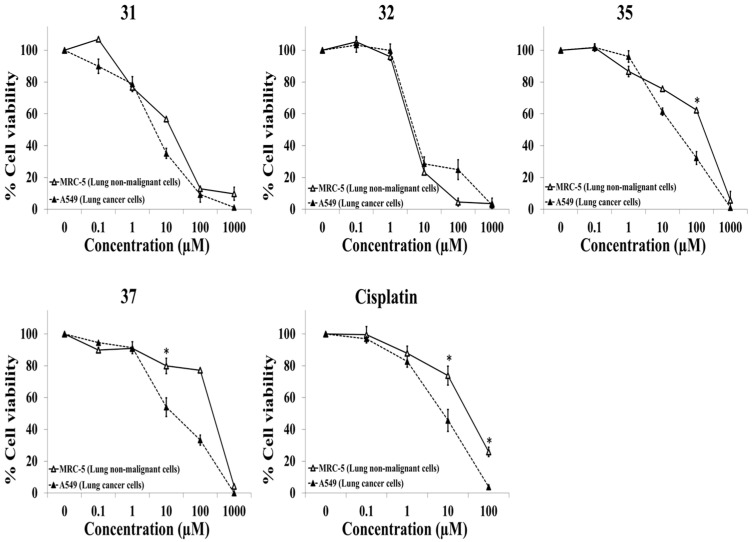
Evaluation of cytotoxic activity of compounds **31**, **32**, **35**, **37**, and cisplatin on human lung nonmalignant cells (MCR-5) and human lung cancer cells (A549). Cells were exposed to several concentrations of compounds for 72 h, and cell viability was determined with the MTT assay. Data represent mean ± SEM from at least two independent experiments. Student’s *t*-test was performed to compare the cytotoxicity of a particular concentration of the compound between MRC-5 and A549. * indicates *p* ˂ 0.05.

Benzofurane scaffold, piperazine ring, and urea function are relevant privileged structures present not only in anticancer drugs against breast cancer but also against other types of cancer, such as lung cancer, hepatocarcinoma, and gastric cancer [20,28,40,41,42,43]. To identify the potential broad-spectrum anticancer activity of our piperazine derivatives, we tested the most selective compounds in non-small cell lung cancer, the second most common cancer worldwide after breast cancer and the leading cause of cancer-related deaths [1]. MRC-5 normal lung cells and A549 lung cancer cells were exposed to compounds **31**, **32**, **35**, **37**, and cisplatin (a common drug used to treat lung cancer) for 72 h, and cell viability was measured using the MTT assay (Figure 10 and Table 3). Compounds **35** and **37** also had high selectivity for A549 cancer cells. While compound **35** showed similar IC_50_ values in A549 and MCF7 cells (25.6 and 23.0 µM, respectively), compound **37** was more cytotoxic against A549 than MCF7 cells (18.7 and 57.1 µM, respectively). It should be mentioned that both compounds showed a slightly better selective profile against lung cancer cells than the anticancer drug cisplatin. On the other hand, compounds **31** and **32** showed lower IC_50_ values against A549 than compounds **35** and **37**, but they were also very toxic for MRC-5 normal cells, showing lower selective profiles.

**Figure 11:**
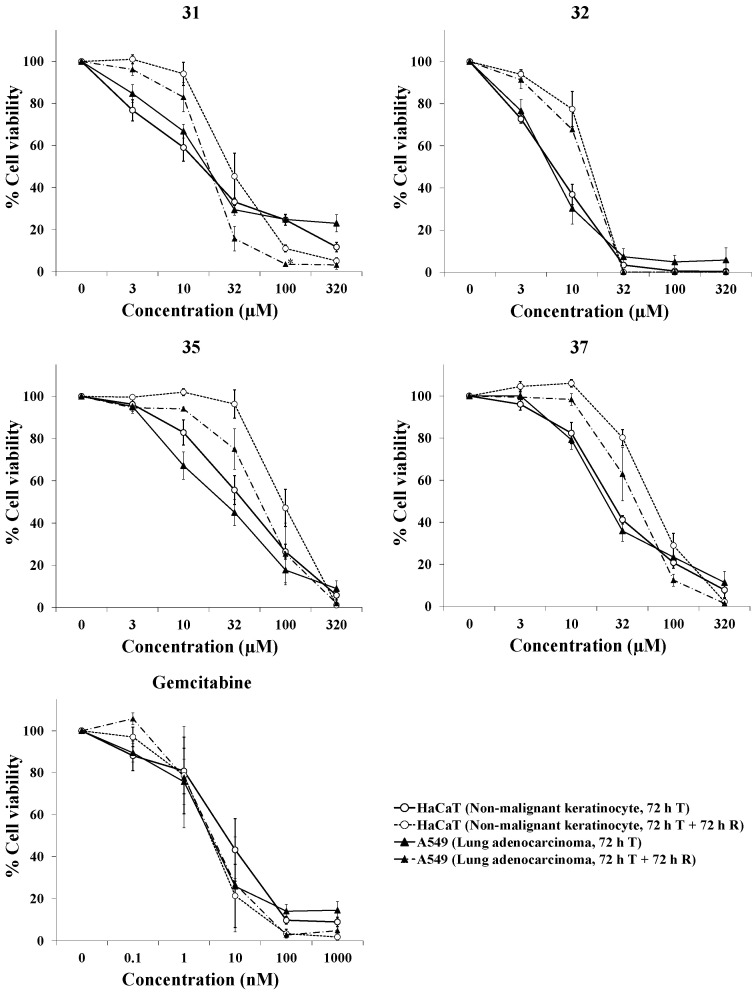
Evaluation of cytotoxic activity of compounds **31**, **32**, **35**, **37**, and gemcitabine on human nonmalignant cells (HaCaT) and human cancer cells (A549). Cells were exposed to several concentrations of compounds for 72 h (“72 h T”, continuous lines), and cell viability was determined with the resazurin assay. Subsequently, the cells were washed and were allowed to grow in drug-free medium for another 72 h (“72 h T + 72 h R”, discontinue lines). Cell viability was again measured with the resazurin assay. Data represent mean ± SEM from at least three independent experiments.

One key limitation of most anticancer drugs is that they not only target cancer cells but also affect nonmalignant cells with high division rates. This can induce numerous adverse effects, such as severe nausea and vomiting, hair loss, diarrhea, mouth sores, anemia, erythematous eruptions, etc. For example, skin cells, which are constantly renewing themselves, are often damaged by antimetabolites or alkylating agents. This can lead to painful erythematous eruptions [44]. Because MCF 10A and MRC-5 are normal cells with a low proliferation rate, we decided to test the most selective compounds on nonmalignant HaCaT cells. These cells are derived from normal adult tissue and have a division rate similar to that of cancer cells. A549 cells and HaCaT cells were exposed to various concentrations of **31**, **32**, **35**, **37**, or gemcitabine (a common antimetabolite used to treat cancer) for 72 h. Cell viability was determined with the resazurin assay. Subsequently, the cells were allowed to grow in a drug-free medium for another 72 h. This was performed to test their survival and proliferation ability after the removal of the compounds. Cell viability was again measured with the resazurin assay. The results are represented in Figure 11 and Table 4. Compounds **32**, **35**, and **37** showed similar IC_50_ values for A549 to those obtained with the MTT assay. Compounds **31**, **35**, and **37** showed selective anticancer activity, although the selectivity profile was lower than against the normal cell lines previously described. A549 cancer cells were approximately two times more sensitive to compounds **31**, **35**, and **37** than nonmalignant HaCaT cells. It should be mentioned that cells exposed to high concentrations of compounds above 100 μM were unable to recover after treatment.

### 2.4. In Silico Evaluation of Physicochemical and Pharmacokinetic Properties of Selected Compounds

In the drug development process, most new drug candidates fail in clinical trials due to their reduced ADME (absorption, distribution, metabolism, and excretion) properties. In silico ADME screens provide a good starting point for selecting the most promising candidates for development and rejecting those with a low chance of success [45]. Selected compounds (**26**–**28**, **31**, **32**, **35**, **37**, and **41**) were tested for drug likeness by Lipinski’s rule of five [46] through the SwissADME online tool (available from URL: http://http://www.swissadme.ch, accessed on 29 November 2023) [47] (Table S1, Supplementary Information). All the selected compounds mirrored Lipinski’s rule. Additionally, compounds with polar surface area (PSA) lower than 140 A^2^ and 10 or fewer rotatable bonds should exhibit high oral bioavailability [48]. Polar surface area is a factor involved in the calculation of percentage absorption (%ABS) being inversely proportional to %ABS by the following equation (%ABS = 109 − (0.345 TPSA) [47], with consequent predicted percentage oral absorption from 70.49 to 87.65% indicating good permeability and transport via biological membranes [49,50]. 

Regarding the ADME properties of the compounds, we decided to analyze some key features that are relevant to the druggability profile of a target compound, such as absorption and distribution, using the Pre-ADMET software, from BMDRC, Seoul, Republic of Korea (available from URL: http://preadmet.bmdrc.org, accessed 29 November 2023) [51,52]. Human intestinal absorption (HIA), Caco-2 cell permeability, skin permeability, blood–brain barrier penetration (BBB), and plasma protein binding (PPB) were predicted using this program. The outcomes of the predicted ADME parameters are shown in Table S2, Supplementary Information.

All the compounds were predicted with a good intestinal absorption above 96% (well-absorbed compounds: 70–100%ABS) and medium cell permeability in the Caco-2 model, with values from 21.80 to 45.10 nm/s (normal range 4–70 nm/s).

BBB is an important predictor for central nervous system (CNS) drug discovery. The obtained results showed two types of CNS access: five compounds (**26**, **28**, **35**, **37**, and **41**) displayed low CNS absorption, with values < 0.1, and three compounds (**27**, **31**, and **32**) gave values ranging from 0.1 to 2.0, exhibiting medium CNS absorption. **31** and **32**, those with Cl and CF_3_ di-substitution patterns (Log P < 4.6), were the most highly absorbed in CNS [49].

Results also showed that six compounds were weakly bound to plasma protein, displaying PPB values of <90%, although three of them (**31**, **32**, and **41**) gave 89.0–89.44 values near enough to be considered strongly bounded. Compounds **35** and **37** displayed values of >90%.

In general terms, these selected compounds presented predicted physicochemical and pharmacokinetic properties that support their consideration as promising hits for the development of a novel class of anticancer agents.

## 3. Materials and Methods

### 3.1. Biology

#### 3.1.1. Drugs and Reagents

3-(4,5-Dimethylthiazol-2-yl)-2,5-diphenyltetrazolium bromide (MTT) was purchased from Panreac Applichem (Darmstadt, Germany). Docetaxel and rezasurin were bought from Sigma-Aldrich (St. Louis, MO, USA). Cisplatin was bought from Thermo Scientific Acros Organics (Waltham, MA, USA). Gemcitabine was obtained from Pfizer S.L. (Madrid, Spain).

#### 3.1.2. Cell Lines

The human A549 lung adenocarcinoma cells and the human lung fibroblasts MRC-5 were purchased from the European Collection of Cell Cultures (Salisbury, UK). The human MCF7 breast adenocarcinoma and human MCF 10A breast epithelial cell lines were generously provided by Dr. D. Ruano and Dr. P. Daza (University of Seville), who obtained the cells from the American Type Culture Collection (ATCC, Manassas, VA, USA) [53]. The human keratinocytes HaCaT cells [54] were purchased from the Cell Line Services (CLS, Hamburg, Germany).

MCF7, A549, MRC-5, and HaCaT were maintained in Dulbecco’s Modified Eagle Medium (DMEM, Biowest, Nuaillé, France) supplemented with 2 mM glutamine (Biowest), 100 μg/mL penicillin (Biowest), 100 μg/mL streptomycin (Biowest), and 10% fetal bovine serum (Biowest). MCF 10A was maintained in a 1:1 mixture of DMEM and Ham’s F12 medium (Biowest), supplemented with 20 ng/mL epidermal growth factor (Calbiochem, Billerica, MA, USA), 100 ng/mL cholera toxin (Sigma-Aldrich, Madrid, Spain), 10 μg/mL insulin (Sigma-Aldrich), 500 ng/mL hydrocortisone (Calbiochem), and 5% horse serum (Gibco, Alcobendas, Spain). Cells were used in low passages after thawing (maximum 14 passages). The cells were cultured in a humidified CO_2_ atmosphere at 37 °C. In general, when cells reached about 80% confluence, the cells were subcultured to ensure proper growth and health.

#### 3.1.3. Cell Viability Assays

Exponentially growing cells were seeded into 96-well plates, and drugs were added 24 h later. Each compound was dissolved with DMSO (Panreac Applichem) to prepare a stock solution (100 mM). The working solutions of specific concentrations were prepared via the dilution of the stock solutions in the culture medium and were immediately used to treat the cells. The remaining stock solutions were aliquoted and frozen at −40 °C. We used different aliquots in each independent experiment to avoid the freeze–thaw cycles. Cells were exposed to the drugs for 72 h. After these 72 h, cell viability was determined with the MTT assay or the resazurin assay. These assays are colorimetric techniques that are widely used to determine cell viability.

The MTT assay is based on the capability of viable cells to transform the MTT salt into a purple formazan dye. After an incubation period of the cells with the MTT and a solubilization step, the quantity of the colored product is measured with a plate reading spectrophotometer. Dead cells are metabolically inactive and cannot produce the colored product. The quantity of formazan produced is presumably proportional to the number of viable cells [55]. After seeding for 24 h, cells were treated with the tested drugs for 72 h. Then, medium was removed, and 125 μL of MTT (1 mg/mL in medium) was added to each well for 3–4 h. Subsequently, 80 μL of 20% sodium dodecyl sulfate (Panreac Applichem) in 20 mM hydrochloric acid (Panreac Applichem) was added to dissolve the insoluble purple formazan product, plates were incubated for 10 h at 37 °C, and optical densities were measured at 540 nm on a multiwell plate spectrophotometer reader.

The resazurin assay measures the ability of viable cells to transform the blue compound resazurin into the pink compound resorufin. There is a direct correlation between the reduction in resazurin and the number of viable cells. After seeding for 24 h, cells were exposed to several concentrations of compounds. After a 72 h treatment period, the cells were washed once with a phosphate-buffered saline (PBS, biowest), and 150 μL of resazurin (20 μg/mL in the medium) was added to each well. The plates were then incubated for 4–5 h at 37 °C, 5% CO_2_, and optical densities were measured at 540 nm and 620 nm using a multiwell plate spectrophotometer reader (Imark Bio Rad Laboratories Inc., Hercules, CA, USA). Subsequently, cells were washed once with PBS and allowed to grow for an additional 72 h in drug-free medium to allow them to recuperate from possible damage induced by the tested compounds. After the treatment period, cell viability was measured following the same protocol described above.

In both assays, cell viability was expressed as percentage in relation to controls. All data were averaged from at least two independent experiments and were expressed as mean ± standard error of the mean (SEM). Selectivity indices (SI) were calculated by dividing the IC_50_ values in the nonmalignant cells by those in the cancer cells [56].

### 3.2. Chemistry

#### 3.2.1. General Methods

All reagents, solvents, and starting materials were obtained from commercial suppliers and were used without further purification. The crude reaction mixtures were concentrated under reduced pressure by removing the organic solvents in a rotary evaporator. Reactions were monitored by thin layer chromatography (TLC) using Kieselgel 60 F_254_ (Merck, Rahway, NJ, USA) plates and UV detector for visualization. Flash column chromatography was performed on Silica Gel 60 (Merck). All reported yields were of purified products. Melting points were obtained on a Stuart Melting Point Apparatus SMP 10 and were uncorrected. Mass spectra were recorded on an Orbitrap Elite (Thermo Scientific, Whaltman, MA, USA), a hybrid ion trap–orbitrap mass spectrometer capable of acquiring a resolution higher than 240,000, with ESI, HESI, APCI, and nanoESI ionization sources. NMR spectra were recorded at 25 °C on a Bruker AV500 spectrometer at 500 MHz for ^1^H and 125 MHz for ^13^C. The chemical shifts (δ) reported are given in parts per million (ppm) on the δ scale relative to TMS, and the coupling constants (J) are in hertz (Hz). ^1^H chemical shift values (δ) are referenced to the residual nondeuterated components of the NMR solvents (δ = 7.26 ppm for CDCl_3_). The ^13^C chemical shifts (δ) are referenced to deuterated solvent (central peak, δ = 77.16 ppm) as the internal standard. The spin multiplicities are reported as s (singlet), d (doublet), t (triplet), q (quadruplet), m (multiplet), or br s (broad singlet). The purity of final compounds was evaluated by elemental analysis (C, H, and N). The purity of all the final compounds was confirmed to be ≥95% by combustion.

#### 3.2.2. Chemoselective N-Acylation Reaction

Synthesis of 1-(benzofuran-2-carbonyl)-3-methylpiperazine (**40**) [15]. 2-Methyl piperazine (6.0 mmol) was dissolved in dry dichloromethane (100 mL) and cooled to 0 °C. A solution of benzofuran-2-carbonyl chloride (6.0 mmol) in dichloromethane (20 mL) was added dropwise for 30 minutes, and then pyridine (10 mmol). The reaction mixture was kept in an ice-water bath with stirring for 6 h and left at room temperature until TLC showed that all the starting material had reacted (2 h). The reaction mixture was evaporated to dryness to obtain the corresponding monoacyl derivative. Flash column chromatography gave the pure compound in high yield. The product was obtained as a solid and purified by column chromatography using dichloromethane–methanol (40:1) as eluent (1.1 g, 74% yield), mp 101−103 °C. ^1^H NMR (500 MHz, DMSO-d_6_) δ 7.7−7.5 (m, 5H), 4.47 (br s, 2H), 3.10 (d, *J* = 11.4 Hz, 1H), 2.94−2.86 (m, 2H), 1.97 (br s, 2H), 1.13 (d, *J* = 5.0 Hz, 3H). ^13^C NMR (125 MHz, DMSO-d_6_) δ 159.8, 154.6, 149.1, 127.0, 126.4, 123.6, 122.2, 111.9, 111.8, 51.1, 46.1, 19.4. Anal. Calcd for C_14_H_17_N_2_O_2_: C, 68.55; H, 6.99; N, 11.42. Found: C, 68.32; H, 6.62; N, 11.22.

#### 3.2.3. Synthesis of 4-(Benzofuran-2-carbonyl)-2-methyl-1-[(1-adamantyl)aminocarbonyl]piperazine (**41**)

To a solution of the monoacyl derivative (**40**) (1.0 mmol) in dry dichloromethane (10 mL) was added the 1-adamantyl isocyanate (1.2 mmol). The reaction mixture was stirred at room temperature until TLC showed that all the starting material had reacted (overnight). The reaction mixture was evaporated to dryness. The product was obtained as a solid and purified by column chromatography using hexane-ethyl acetate (1.7:1) as eluent (0.31 g, 73% yield); mp 183–185 °C. ^1^H NMR (500 MHz, CDCl_3_) *δ* 7.7–7.3 (m, 5H), 4.51 (bs, 1H), 4.36 (dt, *J* = 2.0 Hz, *J* = 13.3 Hz, 1H), 4.2–4.1 (m, 2H), 3.74 (m, 1H), 3.20 (dt, *J* = 3.4 Hz, *J* = 12.9 Hz, 1H), 2.09 (bs, 3H), 2.00 (d, *J* = 2.6 Hz, 6H), 1.68 (t, *J* = 3.2 Hz, 6H), 1.13 (d, *J* = 6.7 Hz, 3H). ^13^C NMR (500 MHz, CDCl_3_) *δ* 160.5, 156.1, 154.7, 148.8, 126.9, 126.7, 123.7, 122.4, 112.6, 111.9, 51.1, 47.5, 42.6, 43.4, 38.6, 36.5, 29.8, 29.6, 29.5, 15.0. HRMS (*m*/*z*): calcd for C_25_H_31_N_3_O_3_Na 444.22576 [M+Na]+; found 444.2251.

## 4. Conclusions

In this study, a collection of 34 compounds of 4-acyl-1-phenylamino(thio)carbonyl-2-substituted piperazine derivatives were evaluated against breast cancer cells, searching for small molecules that can be considered promising anticancer agents. The structural requirements that improve the cytotoxic and selectivity profiles were the central core of 2-pheny-piperazine possessing a Boc group at N-4 and an aryl urea function at N-1 with electron-withdrawing substituents at *para* position of the phenyl ring.

When the 2-methyl-piperazine ring was present, the best activity was observed in those compounds with a benzofuran-2-carbonyl at N-4 instead of the Boc group, and similar electronic properties of the substituents at the phenyl ring in the urea function were needed for the activity. The replacement of the aryl by the adamantyl group in the urea function led to compound **41** having a promising activity/selectivity profile.

From this evaluation, six compounds could be considered an interesting starting point to go deeper into this scaffold, searching for novel small molecules against anti-breast cancer based on the piperazine ring. They were selected based on their activity/security data (**27**, **28**, **31**, and **41** with IC_50_ < 20 µM; **26** and **32** with IC_50_ < 10 µM).

The compounds **31**, **32**, **35**, and **37**, the most selective compounds against breast cancer cells, were also tested in non-small cell lung cancer cells. All compounds decreased the viability of A549 lung cancer cells, which was measured using two different cell viability techniques (MTT and resazurin assays). Two of them, **35** and **37**, showed selective anticancer activity, being less toxic to nonmalignant cells (MRC-5 and HaCaT cells).

Future research will be focused on preserving this general backbone (2-methyl or phenyl piperazine as central core) and introducing chemical diversity not only through the use of different urea and acyl groups but also different organic functions as linkers to generate different analogs with improving biological profiles. It will also study whether these compounds are selective against other types of cancer.

## Figures and Tables

**Table 1:**
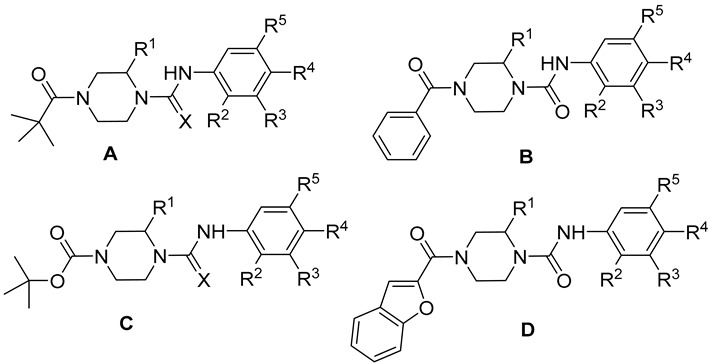
4-Acyl-1-phenylamino(thio)carbonyl-2-substituted-piperazine derivatives.

Compound	Structure	X	R^1^	R^2^	R^3^	R^4^	R^5^
**7**	A	O	CH_3_	H	H	NO_2_	H
**8**	A	O	CH_3_	H	H	OCH_3_	H
**9**	A	S	CH_3_	H	H	NO_2_	H
**10**	B	O	CH_3_	H	H	NO_2_	H
**11**	B	O	CH_3_	H	H	OCH_3_	H
**12**	C	O	CH_3_	H	H	NO_2_	H
**13**	C	O	CH_3_	H	H	OCH_3_	H
**14**	C	S	CH_3_	H	H	NO_2_	H
**15**	C	O	CH_3_	H	H	Cl	H
**16**	C	O	CH_3_	H	H	CF_3_	H
**17**	C	O	CH_3_	H	Cl	CF_3_	H
**18**	D	O	CH_3_	H	H	NO_2_	H
**19**	D	O	CH_3_	H	H	Cl	H
**20**	D	O	CH_3_	H	H	CF_3_	H
**21**	D	O	CH_3_	H	H	CN	H
**22**	D	O	CH_3_	NO_2_	H	H	H
**23**	D	O	CH_3_	OCH_3_	H	H	H
**24**	D	O	CH_3_	Br	H	H	H
**25**	D	O	CH_3_	H	H	H	H
**26**	C	O	Ph	H	H	NO_2_	H
**27**	C	O	Ph	H	H	Cl	H
**28**	C	O	Ph	H	H	CN	H
**29**	C	O	Ph	H	H	F	H
**30**	C	O	Ph	NO_2_	H	H	H
**31**	C	O	Ph	Cl	H	H	CF_3_
**32**	C	O	Ph	H	CF_3_	Cl	H
**33**	C	O	Ph	H	H	OCH_3_	H
**34**	C	O	Ph	H	H	CH_3_	H
**35**	D	O	Ph	H	H	NO_2_	H
**36**	D	O	Ph	H	H	Cl	H
**37**	D	O	Ph	H	H	CN	H
**38**	D	O	Ph	Cl	H	H	CF_3_
**39**	D	O	Ph	NO_2_	H	H	H

**Table 2:** IC_50_ values for compounds **7**–**39** and docetaxel (positive control) in MCF7 breast cancer cells and MCF 10A normal breast cells.

	IC_50_ (Mean ± SEM; μM) ^a^		
Compound	MCF 10A (Normal)	MCF7 (Cancer)	Selectivity Index ^b^
**7**	38.5 ± 4.6	60.8 ± 1.2	0.6 ± 0.1
**8**	540.6 ± 259.1	117.6 ± 48.7	5.2 ± 3.1
**9**	58.2 ± 18.3	15.8 ± 8.2	4.2 ± 1.0
**10**	34.1 ± 1.7	26.7 ± 3.3	1.3 ± 0.1
**11**	477.6 ± 35.6	358.8 ± 74.7	1.4 ± 0.4
**12**	84.2 ± 12.4	34.6 ± 4.8	2.4 ± 0.0
**13**	915.1 ± 226.4	250.5 ± 19.4	3.6 ± 0.6
**14**	35.6 ± 4.5	34.2 ± 0.1	1.0 ± 0.1
**15**	169.9 ± 61.6	43.4 ± 3.7	3.8 ± 1.1
**16**	60.1 ± 19.8	31.3 ± 3.6	1.9 ± 0.4
**17**	41.7 ± 4.1	22.7 ± 1.6	1.9 ± 0.2
**18**	30.4 ± 1.8	12.1 ± 0.6	2.5 ± 0.2
**19**	261.0 ± 2.6	30.5 ± 1.1	8.6 ± 0.4
**20**	243.8 ± 8.2	31.7 ± 4.1	7.8 ± 0.7
**21**	31.2 ± 2.8	19.4 ± 3.8	1.6 ± 0.2
**22**	32.4 ± 1.9	28.1 ± 1.2	1.2 ± 0.1
**23**	74.7 ± 30.2	31.4 ± 5.5	2.3 ± 0.6
**24**	33.5 ± 3.5	25.3 ± 1.6	1.3 ± 0.1
**25**	160.9 ± 64.6	48.5 ± 10.1	3.2 ± 0.7
**26**	71.3 ± 3.6	9.6 ± 6.7	15.2 ± 11.1
**27**	33.8 ± 6.0	16.8 ± 4.0	2.2 ± 0.9
**28**	55.2 ± 24.4	19.5 ± 3.5	2.7 ± 0.8
**29**	56.3 ± 16.5	24.8 ± 1.7	2.2 ± 0.5
**30**	63.2 ± 10.9	24.3 ± 3.4	2.6 ± 0.1
**31**	297.7 ± 56.4	18.6 ± 4.2	16.6 ± 1.9
**32**	34.1 ± 2.2	2.7 ± 0.6	12.6 ± 2.6
**33**	325.0 ± 27.6	130.4 ± 31.4	2.6 ± 0.4
**34**	68.7 ± 2.8	24.4 ± 3.1	2.9 ± 0.5
**35**	244.4 ± 24.9	23.0 ± 3.8	11.1 ± 2.9
**36**	160.8 ± 16.5	41.3 ± 4.2	4.0 ± 0.9
**37**	484.2 ± 233.0	57.1 ± 34.7	17.9 ± 7.9
**38**	116.9 ± 21.1	91.9 ± 35.7	1.4 ± 0.3
**39**	>1000	225.9 ± 175.0	>2.5
**Docetaxel** (nM)	˂1	76.1 ± 26.2	˂1

^a^ Data averaged from at least two independent experiments are expressed as mean ± standard error of the mean (SEM). ^b^ Selectivity index was calculated by dividing the IC_50_ value obtained in the nonmalignant cell line by that in the cancer cell line.

**Table 3:** IC_50_ values for compounds **31**, **32**, **35**, **37**, and cisplatin (positive control) on human lung nonmalignant cells (MCR-5) and human lung cancer cells (A549).

	IC_50_ (Mean ± SEM; μM) ^a^		
Compound	MRC-5 (Normal)	A549 (Cancer)	Selectivity Index ^b^
**31**	14.2 ± 0.5	4.8 ± 0.8	2.1 ± 0.3
**32**	4.3 ± 0.2	5.2 ± 0.4	1.0 ± 0.1
**35**	165.4 ± 4.1	25.6 ± 2.9	6.5 ± 2.1
**37**	235.7 ± 5.4	18.7 ± 2.7	12.8 ± 1.6
**Cisplatin**	31.8 ± 5.6	8.2 ± 2.2	6.3 ± 1.8

^a^ Data averaged from at least two independent experiments are expressed as mean ± standard error of the mean (SEM). ^b^ Selectivity index was calculated by dividing the IC_50_ value obtained in the nonmalignant cell line by that in the cancer cell line.

**Table 4:** IC_50_ values for compounds **31**, **32**, **35**, **37**, and gemcitabine (positive control) on human nonmalignant cells (HaCaT) and human cancer cells (A549).

	72 h Treatment			72 h Treatment + 72 h Recovery Period		
	IC_50_ (Mean ± SEM; μM) ^a^			IC_50_ (Mean ± SEM; μM) ^a^		
Compound	HaCaT (Nonmalignant)	A549 (Cancer)	Selectivity Index ^b^	HaCaT (Nonmalignant)	A549 (Cancer)	Selectivity Index ^b^
**31**	14.5 ± 3.2	17.0 ± 1.6	0.8 ± 0.2	30.9 ± 7.5	17.8 ± 2.0	1.8 ± 0.5
**32**	6.7 ± 0.8	6.2 ± 0.6	1.1 ± 0.1	14.9 ± 1.3	13.6 ± 0.1	1.1 ± 0.1
**35**	41.6 ± 8.1	28.4 ± 7.3	1.6 ± 0.2	97.5 ± 16.2	69.3 ± 20.2	2.0 ± 0.8
**37**	24.8 ± 1.8	22.5 ± 2.7	1.1 ± 0.1	64.6 ± 6.8	41.6 ± 8.9	1.9 ± 0.6
**Gemcitabine** (nM)	13.1 ± 6.2	3.5 ± 0.8	3.3 ± 1.4	8.8 ± 6.5	9.3 ± 7.0	1.0 ± 0.1

^a^ Data averaged from at least three independent experiments are expressed as mean ± standard error of the mean (SEM). ^b^ Selectivity index was calculated by dividing the IC_50_ value obtained in the nonmalignant cell line by that in the cancer cell line.

## Data Availability

Data are contained within the article and Supplementary Materials.

## Supplementary Materials

The following supporting information can be downloaded at: https://www.mdpi.com/article/10.3390/ijms242317041/s1.
