# Supplementary material for: Repurposing Study of 4-Acyl-1-phenylaminocarbonyl-2-substituted-piperazine Derivatives as Potential Anticancer Agents—In Vitro Evaluation against Breast Cancer Cells
Source: Int J Mol Sci. 2023 Dec 1;24(23):17041. doi: 10.3390/ijms242317041 (PMC10706865; doi:10.3390/ijms242317041)
Supplement: Supplementary file 1 [file ijms-24-17041-s001.zip › ijms-2727374-supplementary.pdf]

Repurposing study of 4-Acyl-1-phenylaminocarbonyl-2-substituted-piperazine derivatives as potential antitumoral agents. *In vitro* evaluation against breast cancer cells.

Emilio Guillén-Mancina,† María del Rosario García-Lozano,† Estefanía Burgos-Morón, Sarah Mazzotta, Pablo Martínez-Aguado, José Manuel Calderón-Montaño, José Manuel Vega-Pérez, Miguel López-Lázaro, Fernando Iglesias-Guerra* and Margarita Vega-Holm*

CONTENTS

Figure S1 Evaluation of cytotoxic activity of compounds **7**, **8**, **11 - 17** on human non-malignant breast cells (MCF 10A) and human breast cancer cells (MCF7).

Figure S2 Evaluation of cytotoxic activity of compounds **21** - **25** on human non-malignant breast cells (MCF 10A) and human breast cancer cells (MCF7).

Figure S3 Evaluation of cytotoxic activity of compounds **27** – **30**, **33** and **34** on human non-malignant breast cells (MCF 10A) and human breast cancer cells (MCF7).

Figure S4. Evaluation of cytotoxic activity of compounds **36**, **38** and **39** on human non-malignant breast cells (MCF 10A) and human breast cancer cells (MCF7).

Figure S5 NMR-Spectra of compound **41**

Figure S6 HRMS spectra compound **41**

**Table S1**	Predicted physicochemical properties of selected compounds using SwissADME software.
**Table S2**	ADME data of selected compounds calculated using preADMET software.

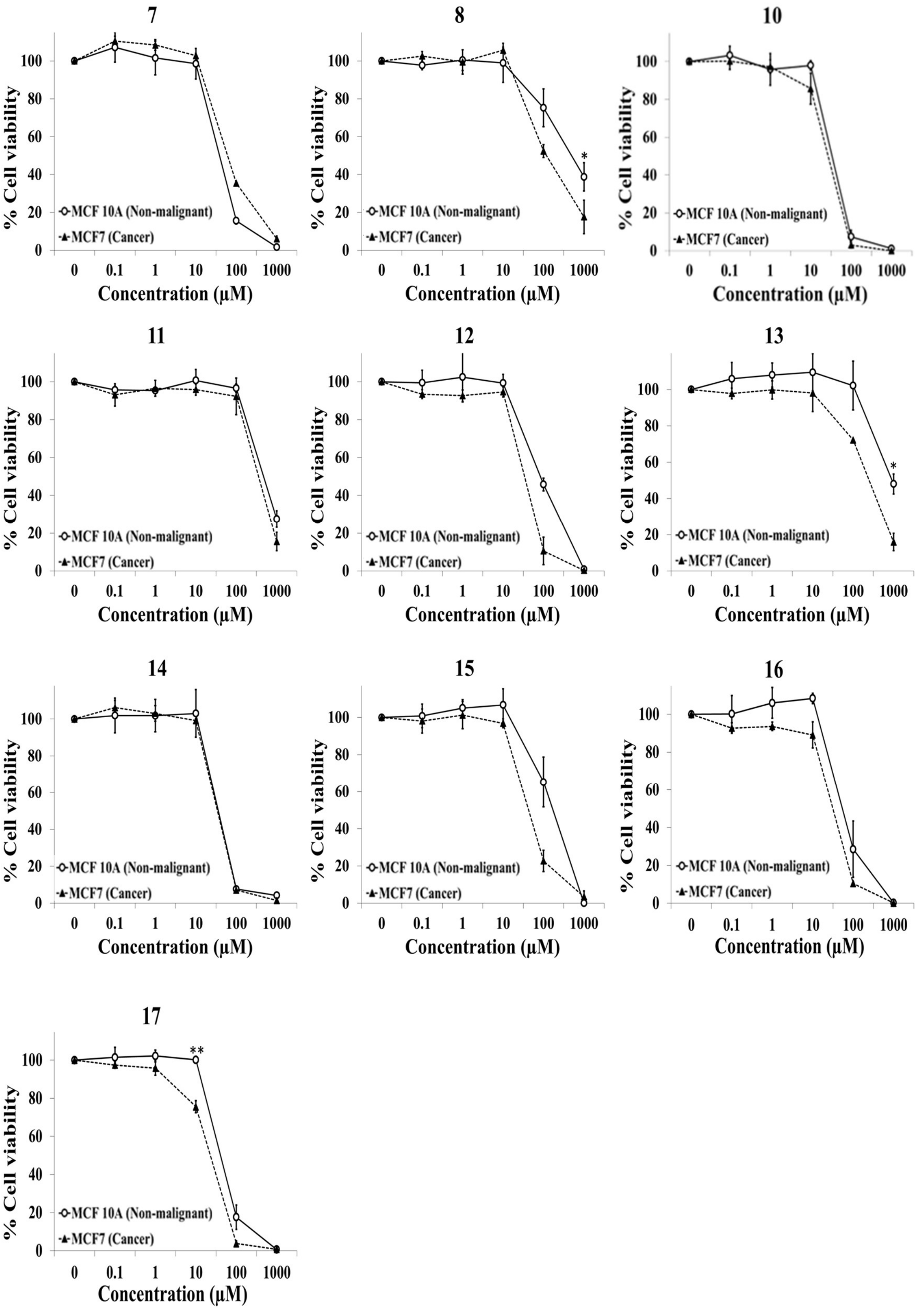

Figure S1. Evaluation of cytotoxic activity of compounds **7**, **8**, **11** - **17** on human non-malignant breast cells (MCF 10A) and human breast cancer cells (MCF7). Cells were exposed to several concentrations of compounds for 72 h and cell viability was determined with the MTT assay. Data represent mean ± SE) from at least two independent experiments. Student's t-test was performed to compare the cytotoxicity of a particular concentration of the compound between MCF 10A and MCF7, * indicates $p < 0.05$, ** indicates $p < 0.01$.

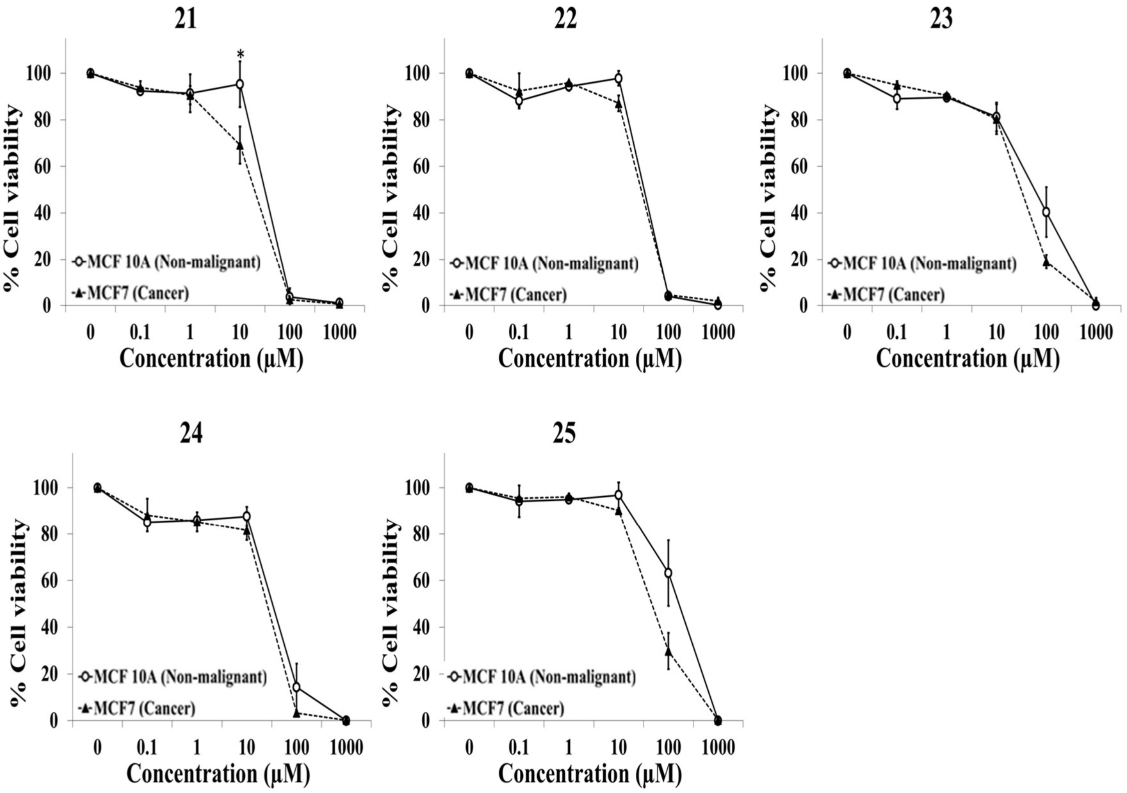

Figure S2. Evaluation of cytotoxic activity of compounds **21** - **25** on human non-malignant breast cells (MCF 10A) and human breast cancer cells (MCF7). Cells were exposed to several concentrations of compounds for 72 h and cell viability was determined with the MTT assay. Data represent mean ± SE) from at least two independent experiments. Student's t-test was performed to compare the cytotoxicity of a particular concentration of the compound between MCF 10A and MCF7, * indicates $p < 0.05$.

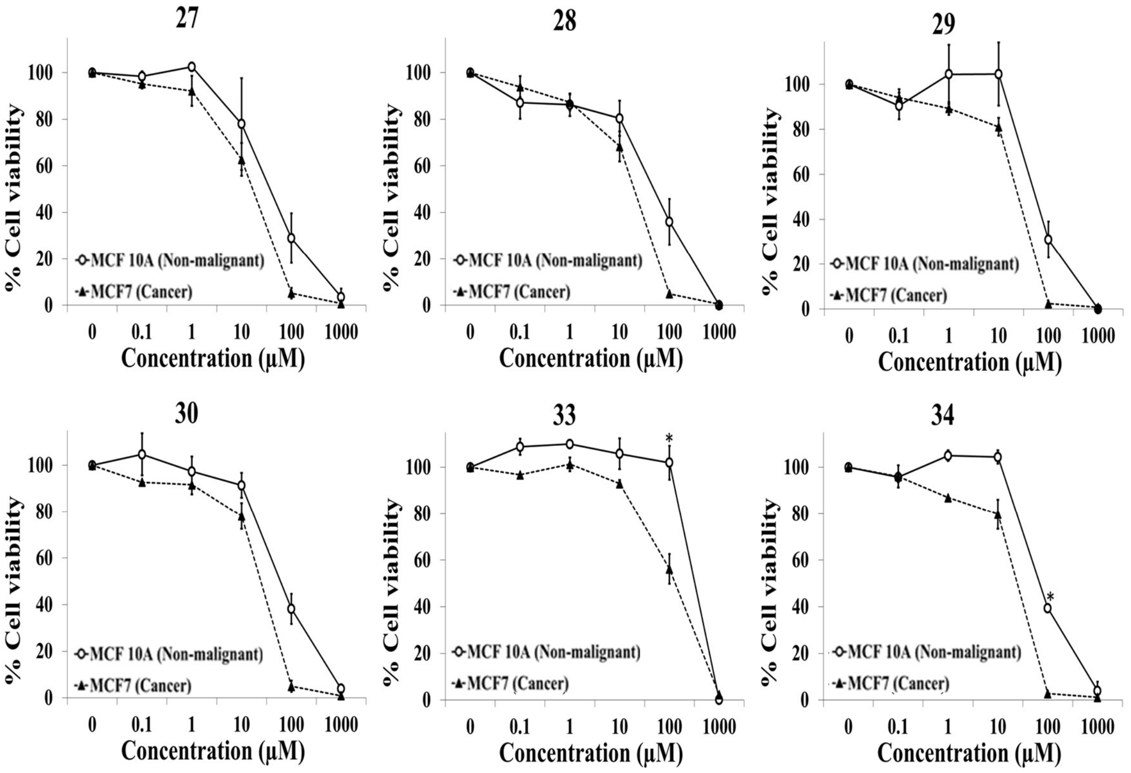

Figure S3. Evaluation of cytotoxic activity of compounds **27** – **30**, **33** and **34** on human non-malignant breast cells (MCF 10A) and human breast cancer cells (MCF7). Cells were exposed to several concentrations of compounds for 72 h and cell viability was determined with the MTT assay. Data represent mean ± SE) from at least two independent experiments. Student's t-test was performed to compare the cytotoxicity of a particular concentration of the compound between MCF 10A and MCF7, * indicates $p < 0.05$.

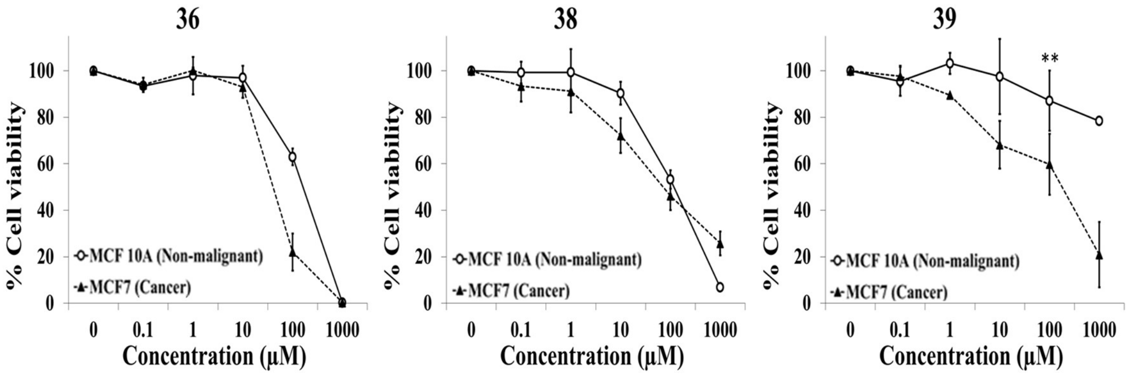

Figure S4. Evaluation of cytotoxic activity of compounds **36**, **38** and **39** on human non-malignant breast cells (MCF 10A) and human breast cancer cells (MCF7). Cells were exposed to several concentrations of compounds for 72 h and cell viability was determined with the MTT assay. Data represent mean ± SE) from at least two independent experiments. Student's t-test was performed to compare the cytotoxicity of a particular concentration of the compound between MCF 10A and MCF7, ** indicates $p < 0.01$.

a)

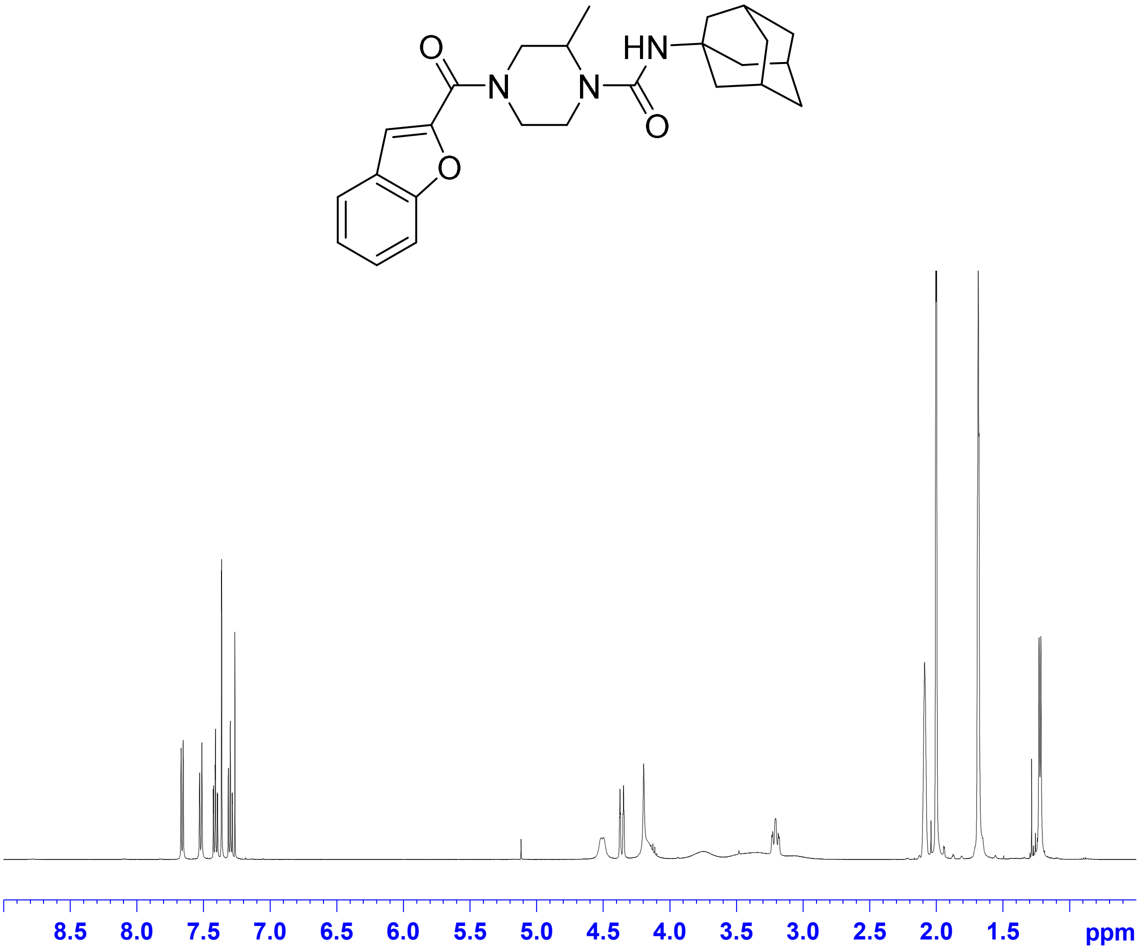

b)

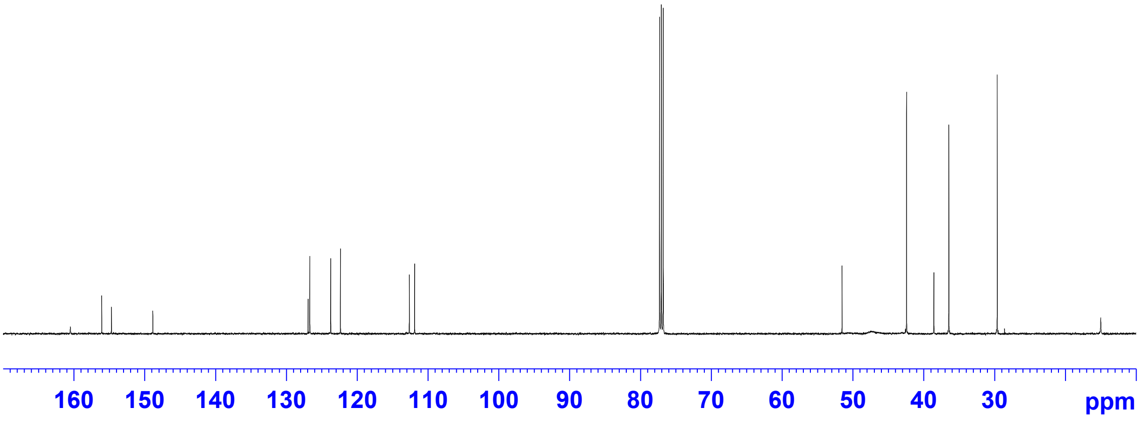

Figure S5. NMR-Spectra of compound 4-(Benzofuran-2-carbonyl)-2-methyl-1-[(1-adamantyl)-aminocarbonyl]piperazine **41. a)** ^{1}H NMR and **b)** ^{13}C NMR.

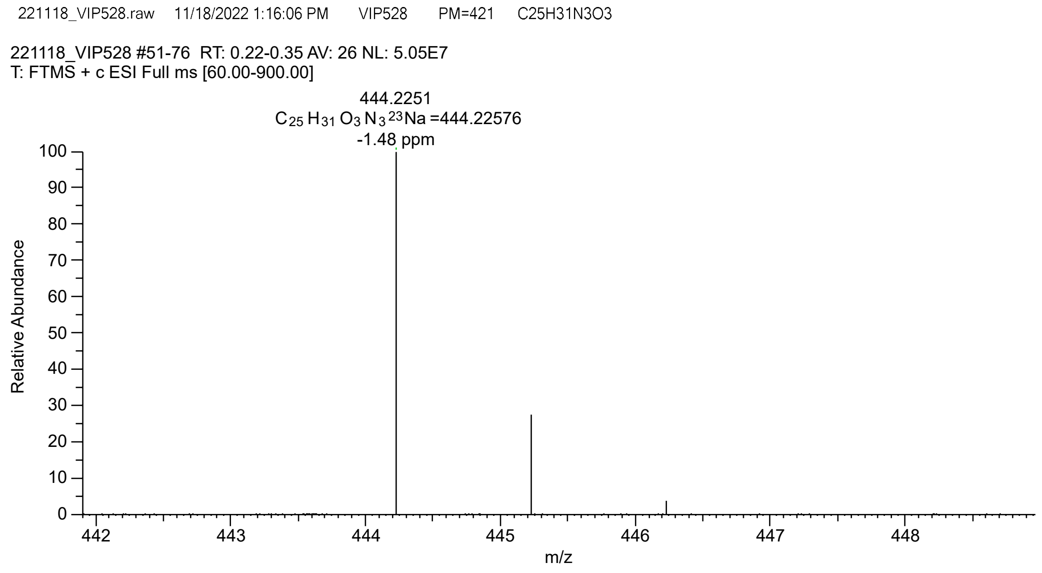

Figure S6. HRMS spectra of 4-(Benzofuran-2-carbonyl)-2-methyl-1-[(1-adamantyl) aminocarbonyl]piperazine (**41**).

Table S1. Predicted physicochemical properties of selected compounds using SwissADME software.

Comp	Parameter							
	**MW**	**LogP**	**HBD**	**HBA**	**nVs**	**TPSA**	**%ABS**	**nrotb**
**AR**	≤500	≤5	≤5	≤10	≤1	<140	-	≤10
**26**	426.47	2.34	1	5	0	107.78	71.82	8
**27**	415.91	3.61	1	3	0	61.88	87.65	7
**28**	406.48	2.86	1	4	0	85.67	79.44	7
**31**	483.91	4.62	1	6	0	61.88	87.65	8
**32**	483.91	4.62	1	6	0	61.88	87.65	8
**35**	470.48	3.06	1	5	0	111.61	70.49	7
**37**	450.49	3.40	1	4	0	89.58	78.09	6
**41**	421.53	3.49	1	3	0	65.79	86.30	5

AR, accepted range; MW, Molecular weight; LogP, lipophilicity, (consensus Log $P_{O/W}$); HBD, Number of hydrogen bond donors; HBA, Number of hydrogen bond acceptors; nVs, Number of Lipinski rule violations; TPSA, PSA ($Å^2$); %ABS, percentage of oral absorption; nrotb, number of rotable bonds.

Table S2. ADME data of selected compounds calculated using preADMET software.

Parameter	Compound							
	**26**	**27**	**28**	**31**	**32**	**35**	**37**	**41**
**Absorption**								
**HIA (%)**	96.43	96.37	96.87	96.49	96.49	97.72	96.69	96.20
**Caco2**	21.80	41.10	23.47	45.10	45.10	22.95	30.15	37.92
**Log Kp**	-2.07	-2.15	-2.09	-1.72	-1.72	-2.49	-2.55	-4.23
**Distribution**								
**BBB**	0.015	0.380	0.036	1.180	1.180	0.039	0.042	0.099
**PPB (%)**	87.11	88.63	86.64	89.06	89.06	91.42	93.23	89.44

HIA: percentage human intestinal absorption; Caco2: permeability through cells derived from Human Colon Adenocarcinoma (nm/s); Log Kp: skin permeability; BBB: blood-brain barrier; PPB: plasma-protein binding.
